# A qualitative study of health promotion in academy schools in England

**DOI:** 10.1186/s12889-019-7510-x

**Published:** 2019-08-28

**Authors:** Patricia E. Jessiman, Rona Campbell, Russ Jago, Esther M. F. Van Sluijs, Dorothy Newbury-Birch

**Affiliations:** 1 Department of Population Health Sciences, University of Bristol, Canynge Hall, 39 Whatley Road, Bristol, BS8 2PS UK; 2 Centre for Exercise, Nutrition & Health Sciences, School for Policy Studies, University of Bristol, 8 Priory Road, Bristol, BS8 1TZ UK; 3 Centre for Diet and Activity Research (CEDAR), MRC Epidemiology Unit, University of Cambridge, Cambridge Biomedical Campus, Cambridge, CB2 0QQ UK; 4 School of Social Sciences, Humanities and Law, Teesside University, Centuria Building, Middlesborough, Tees Valley TS1 3BX UK

**Keywords:** Health, Education, School, Children, Adolescence, Qualitative

## Abstract

**Background:**

Schools are an important setting for health promotion. In England, around one third of publicly funded schools have become independent of local authorities since 2000 and are now academies, run by an academy trust. The aim of this research was to examine attitudes towards health promotion held by academy trust leaders and senior staff. The research questions were: 1. How do academy trusts in England perceive their role in health promotion amongst students? 2. How are decisions around health promotion made in academy trusts? 3. What factors inhibit and encourage health promotion in academy schools? 4. How might public health academics and practitioners best engage with academy schools to facilitate health promotion activity and research?

**Methods:**

Qualitative study utilising semi-structured interviews. Twenty five academy and school leaders were purposively sampled to achieve variation in trust size and type. In addition, five respondents were recruited from public and third-sector agencies seeking to work with or influence academy trusts around health promotion. Framework analysis was used to determine emergent themes and identify relationships between themes and respondent type. Early findings were triangulated at a stakeholder event with 40 delegates from academia, local authority public health teams, and third sector organisations.

**Results:**

There is wide variation amongst senior academy and trust leaders in how they perceive the role of academies in promoting health and wellbeing amongst students. There is also variability in whether academy trusts responsible for more than one school adopt a centralised strategy to health promotion or allow individual schools autonomy. This was dependent on the trust leaders’ attitude and interest in health promotion rather than any perceived external accountability. Identified barriers to health promotion include financial constraints, a narrow focus on educational outcomes and school performance, and limited understanding about effective health interventions.

**Conclusion:**

In the current absence of national policy or guidance around health promotion in schools, health has variable status in academies in England. There is a need to better engage all academy trusts in health promotion and support them to implement a strategic approach to health promotion.

**Electronic supplementary material:**

The online version of this article (10.1186/s12889-019-7510-x) contains supplementary material, which is available to authorized users.

## Background

Schools are key settings for health promotion and healthy children and young people obtain better educational outcomes which, in turn, are associated with better life long health [1]. Worldwide in 2015, 91% of primary-school-age children, and 84% of lower-secondary-aged children, were enrolled in school [2]. In England, schools provide curriculum time for education about sexual health, drugs, alcohol and smoking via mandated aspects of the curriculum. Schools also provide food and a setting for physical activity via physical education as well as trained teachers who are ideally placed to deliver behaviour change and prevention programs to children and adolescents. Schools provide an opportunity to progressively build educational content as children age, adding to the knowledge accumulated in previous years. As such, schools and the concept of a health promoting school are key aspects of education for children and young people that have been supported globally [3]. This holistic approach involves not only health education via the curriculum but also having a school environment and ethos that is conducive to health and wellbeing, and by engaging with families and the wider community, recognising the importance of this wider environment in supporting children and young people’s health. A Cochrane review of the health promoting schools approach, which included 67 randomised controlled trials, concluded that it was effective in improving aspects of student health [4].

School-based health promotion requires curriculum content, guidance and policies on how to ensure a healthy environment, and the support of staff to deliver the content. Traditionally in England this area level support would be provided by the National Health Service (NHS) in collaboration with local authorities which oversaw all state-maintained schools. The National Healthy Schools Programme was introduced to promote a whole school approach to health in England by the UK Department of Health and the Department of Children, Schools and Families in 1998. State-funded schools were supported to achieve National Healthy School Status by Healthy Schools teams located in NHS Primary Care Trusts, but funding for the programme ended in 2011 and responsibility for public health work transferred from the NHS to local authorities, many of whom reduced their support to schools [5]. Around the same time, it became central government policy to substantially increase the number of academy schools.

Academy schools are publicly-funded state schools but are independent of local authorities and receive their funding directly from the UK Department of Education. The academies programme began in England in 2000 under the Labour government as a means of addressing underperforming local authority-maintained secondary schools through the provision of sponsorship by an external partner from sectors including business and industry, philanthropy, universities, religious groups, charities and others including other high performing schools. The coalition government of Conservatives and Liberal Democrats expanded the remit of the programme in 2010 to include high performing primary, secondary, and special educational needs or disabilities (SEND) schools as well as pupil referral units (PRUs) and 16+ institutions who could opt out of local authority control by applying to convert to academy status [6]. An additional file provides an account of academy and school-types in England (see Additional file 1). In addition, schools which are judged as inadequate by the Office for Standards in Education, Children’s Services and Skills (Ofsted; the government agency that inspects all maintained and academy schools in England) are directed by the Department for Education to convert to academy status with the support of a sponsor [7]. As a result the number of sponsored and converted academies has steadily increased to the point where now over a third of schools in England are academies and these schools teach around half of all pupils [7, 8]. In 2018 the proportion of schools within any single local authority that were academies ranged from 6 to 93% [7].

Academies serve both primary and secondary aged-children and are run by an academy trust, which controls the school budget and employs school staff. These can be single academy trusts (SATs) comprising one school, or multi-academy trusts (MATs). The location of schools within MATs can cross local authority boundaries. Academies do not have to follow the English National Curriculum and have some freedom to vary term times [9].

The debate around academies in England has focused largely around whether they are achieving their original aim of raising school standards [6, 10, 11], concern about their democratic accountability, [12, 13], and rising remuneration packages afforded to trust and school leaders [14]. Little attention has been paid to their influence over the health of students and staff. Given the large numbers of students enrolled, and the associated staff they employ, academy trusts are an important setting for health promotion. However, their independence from local authorities and the retrenchment of local healthy schools teams means that health promotion across academy trusts is not currently coordinated and little is known about how academy trusts view their role. This study is concerned with the approach to health promotion adopted by academy trusts.

### Research questions

The aim of this research project was to examine attitudes towards health promotion held by academy trust leaders and senior staff in academy schools. This included, but was not limited to, their perceptions of the health challenges facing their students, and the role of the academy trust in ameliorating these challenges. We were also interested in their attitudes towards undertaking new research into health promotion in schools. The research questions for the current study were:

1. How do academy trusts in England perceive their role in health promotion amongst students?
2. How are decisions around health promotion made in academy trusts?
3. What factors inhibit and encourage health promotion in academy schools?
4. How might public health academics and practitioners best engage with academy schools to facilitate health promotion activity and research?

## Methods

### Semi-structured interviews

A qualitative approach was adopted using semi-structured interviews with elite participants. While there is no agreed definition of who qualifies as an elite interviewee [15] target participants in the study included leaders of MATs and SATs, head teachers and senior staff of academy schools who have considerable power and influence over decision making within that organisation. Elite interviews present challenges because the interviewees’ time is often closely guarded by gatekeepers. Unlike many research interviews where the power largely rests with the questioner, elite interviewees are used to being in charge of situations and questioning other people for their views or to seek information from them. Thus, an elite interviewee may want to dominate the interview and ask questions of the interviewer [16]. It is therefore important that the research interviewer is able to convince both gatekeepers and the prospective interviewee of the merits of being interviewed by explaining clearly what is in it for the interviewee [17]. In addition, the interviewer needs to be knowledgeable about the interviewee’s organisation and role in it [15, 18]. Therefore, a key element of the methodology was gathering as much publicly-available information about the trust and the school prior to interview so that the interviewer (PJ) was prepared with this knowledge and avoid interviewees being asked for information already in the public domain.

Semi-structured interviews allow for structure, flexibility, and flow, ensuring that the interviewer addresses the research questions in full, prompting and probing respondents for further information where necessary. They also allow the respondent to feel engaged in a conversation rather than answering a structured survey. Accordingly, the order of topics, and specific wording of questions asked, varied between interviews but the research questions were addressed in full in each using a topic guide as an aide-memoir for the interviewer. This was developed for this study following an initial literature review of health promotion in schools and of the academies programme and refined following the advice of public health and educational experts on the advisory group for the study. It covered the following main areas: *1. Attitude towards health promotion in schools*; *2. Health promoting initiatives in the academy or trust* including questions around school or trust strategic approach to health promotion; and *3.Undertaking health research* including use of research evidence around health and education, and attitudes towards undertaking new health research in schools. The topic guide (Additional file 2) was piloted with an initial participant and final small refinements made before use with the remaining participants.

### Sampling and recruitment strategy

We developed a purposeful sampling strategy that included representation from trusts of varying sizes (from single schools to large MATs of over 20 schools) and school type (including representation from primary, secondary, faith academies and free schools) across England. One participant from a school federation was also recruited. School federations are formed when maintained schools come together under a single governance body that sets the strategic direction for the group [19]. While federated schools are still under local authority control, we were interested to determine if a federation acted in a similar manner as an academy trust might in relation to health promotion. In order to examine their experiences of health promotion in the academy context, a second, small sample of participants was recruited from public and third-sector agencies seeking to work with or influence academy trusts around health promotion.

The research team began initial recruitment of participants from academy trusts by contacting those with whom they had had contact with previously either through research projects in schools, common membership of committees or advisory groups, or other less formal contact (e.g. chance meetings at conferences and events). Recruitment was then ‘snowballed’ by asking those participants who agreed to be interviewed if they could introduce the study and the researcher to other colleagues in academy trusts. In addition, to ensure our sampling strategy was fulfilled the lead researcher contacted trust leaders without prior introduction using contact details available on their websites.

Participants outside academy trusts were recruited with the help of a notice on regional bulletins issued by Public Health England (an executive agency of the Department of Health and Social Care) in which interested participants were invited to contact the lead researcher. The researcher also contacted several third-sector agencies seeking to establish health-promoting activities in academy schools by email, inviting them to participate.

An invitation to participate was sent by email, alongside a participant information sheet explaining context underpinning the study, and the subject nature of the interview. Participants were informed that the interview would take around 60–90 min, would be digitally recorded for later analysis, and that they could decline to respond to any individual question or withdraw from the interview at any time. They were also informed that the data collected would be anonymised and their participation kept confidential, with anonymised direct quotes possibly used in publicly available reports and other outputs. Respondents were asked to sign a form consenting to each of these points prior to interview. Almost all interviews were undertaken face-to-face at the respondent’s place of work with one researcher; two were undertaken by telephone. Fieldwork took place between March and October 2018.

### Analysis

All interviews were transcribed verbatim, and underwent thematic analysis using the Framework Method [20, 21]. The lead researcher read the transcripts to familiarise herself with the data, and made analytical notes to inform the next, coding stage. During coding, a selection of transcripts were read line by line and initial labels or ‘codes’ applied to each passage that described the essential meaning of the data within. After four transcripts were coded, the researcher compared them to develop an initial thematic framework that identified the main themes and subthemes relating to the research questions. The draft thematic framework was tested and refined with four more transcripts, ensuring that the framework encompassed all the data in the transcripts relevant to the research questions but did not over-simplify. The procedure also ensured that that data within each subtheme was coherent, and that there were clear distinctions between subthemes. The framework was revised in discussion with the wider project team until we were satisfied that it ‘fitted’ the data. The revised framework is shown in Table 1. Once refined, the framework was applied to all transcripts, populating a matrix framework with verbatim and summarised data from the transcripts using NVivo software (‘charting’). Ongoing charting of each interview transcript during and after this process, comparing new data with earlier transcripts, ensured that the resulting matrix provided a detailed and accessible overview of the data populating each theme and subtheme from every respondent. This resulting matrix afforded the possibility of exploring the data by both theme, and respondent-type by all members of the research team, allowing them to analyse the data including developing a description of each theme and subtheme, and move up the ‘analytical hierarchy’ to develop an explanatory analysis including detecting patterns and associations between themes in the data [22, 23].

**Table 1:** Thematic Framework

Theme	Sub-themes
School of role in health promotion	Link between health and attainment Health domains of concern – students Health domains of concern – staff Role of schools/Trusts in health promotion
Decision-making	Health strategy or policy development Health strategy or policy implementation Locus – academy school, Trust, or MAT executive Health budget Health in strategic planning Implementing change across schools and MATs Use of research evidence (and source) Accountability for health promotion activity
Health-promoting initiatives undertaken in schools and Trusts	Domain (e.g. student mental health; obesity, risk behaviours) Curriculum School environment Staff training Ethos Staff/pupil relationship Staff health initiatives Involvement of parents and wider community Involvement of students Facilitators to implementing initiatives Barriers to implementing initiatives Outcomes of initiatives
Links with external agencies	Delivering initiatives in schools/Trusts Health professionals in schools/Trusts Links with Public Health teams Links with statutory health services Other health-related services Healthy schools audit
Health data	Existing/historical health data collected by school/Trust Data from external bodies e.g. local authority, NHS, Public Health England Use of data Missing/inaccessible health data would like to have
Networks	MAT (within and across MATs) School to school Informal networking Local authority links and networks Other networks around health promotion
Drivers for health promotion	Motivating factors for health promotion activity Barriers to undertaking health promotion
Undertaking health research	Experience of working with academia/public health researchers Motivation for involvement in research Barriers to undertaking research Facilitators for undertaking research

Early findings from the study were presented to a group of 40 stakeholders from academia, local authority public health teams, and third sector organisations with experience in working with both maintained and academy schools. A written record of participants’ feedback on these findings, as well as group discussion of their own experience of engaging academy trusts, afforded triangulation of the data.

## Results

### Sample

Thirty respondents completed an in-depth interview with the lead researcher. Table 2 describes limited participant characteristics (to protect anonymity). Twenty-four were from academy trusts and one was the lead of a small federation. The academies within the trusts included a varying range of schools including primary, secondary, faith, free, and special schools. Three education-sector participants were leaders of faith schools, and one led a free school (other MATs in the sample were also responsible for some free and faith schools). The sample includes trust Chief Executive Officers (CEO), head teachers, and those to whom leadership for wellbeing of students and staff has been delegated. In addition, five non-education sector staff were interviewed. These were individuals whose job role included working with schools on health improvement and education, including two staff from local authority public health teams and three from third-sector health organisations.

**Table 2:** Participant Characteristics

Academy school/trust participants (Denoted in quotations as AS)		Participants (*N* = 25)
*Characteristic*	Category	
Trust Size	SAT	5
	Small-medium MAT (≤ 20 schools)	15
	Large MAT (> 20 schools)	4
*Federation*		1
Role	CEO	9
	Deputy CEO	1
	Trustee/Director	3
	Other member of Executive Team	3
	Head teacher	3
	Assistant head teacher	3
	School Wellbeing Lead	3
Non-school/trust participants (Denoted in quotations as NS)		Participants (*N* = 5)
Role	Local Authority Public health officer	2
	Leader of third-sector health organisation	3

The findings are presented according to the four overarching research questions. An additional theme that emerged from the transcripts was respondents’ perceptions of the main health challenges facing students. Anonymised quotations are included from a wide range of participants which serve to illustrate the responses rather than indicate representativeness.

### Key health challenges facing students

Education respondents approached unanimity in their view that the biggest health challenge facing students was to their mental health, and attributed this to factors challenging students both outside and within the school itself. These included those sited within children’s homes, such as domestic violence, criminality, substance misuse, parental unemployment, poverty, and child protection concerns. Community-level factors included the impact of austerity on local services, as well as high rates of unemployment, criminality, anti-social behaviour and substance misuse. Many respondents mentioned social media (mis)use as a key challenge to children’s wellbeing. The role of the education system in contributing to student mental ill health was also acknowledged, which for some was not being fully recognised or addressed.

*We’re talking about the mental health of children, when I think in many respects we’re talking about why schools have projected various pressures onto children over the last 15 years. And that pressure has created a particular response. And rather than talk about the pressure we’re talking about the response to the pressure, which in some ways is frustrating.(AS26).*

Other key challenges facing student health raised by academy trust respondents included obesity, poor nutrition, physical inactivity, risk behaviours (in particular substance misuse) and sleep deprivation. For some, tackling health inequalities was a priority. Those from MATs often talked about the health inequalities that were apparent between the academies in their trust.

*Our most deprived areas are actually [Area A]. Now, what concerns us there is children’s nutrition but also their wellbeing – their mental health…[]…Actually, what’s quite obvious to us is when we do collaborative trips together…the physical stature of the children [from Area A] is quite marked against, maybe, the children [from other areas].* (AS13).

One non-school respondent suggested that identifying and addressing health inequalities was less likely to happen in larger, national MATs working across local authority boundaries, as trust leaders were less engaged with any one local authority area.

*The one that I think [City X] education has had the most problem with is [MAT*] *because they are national so they’re up and down the country…[]…the thing that all [City X] schools cannot get away from is the inequality [here], heads understand that and have the joint responsibility for it. [MAT*] *never seem to do that. You could wave local health statistics at them but they weren’t interested.* (NS28).

### Research question 1: how do academy trusts in England perceive their role in health promotion amongst students?

All academy trust respondents acknowledged there was a link between student health and educational attainment, but they varied in their view of the role of academies in promoting health and wellbeing amongst students. Responses clustered into four categories. A small number felt that academies had no responsibility for health promotion amongst students. A second category of response emerged where respondents held a very functional view of health as a key driver of student attendance, and that academies should promote good attendance as a means of raising educational outcomes for students. All respondents who held views in these two first response categories were executive members of the trust (CEO or other executive post). A third, more populated response category was a perception of health promotion as essential to remove barriers to learning faced by many students. Respondents in this category, who were a mix of staff at the trust executive level as well as school-based staff, tended to focus on poor mental health as a key challenge facing many students. Their view was that academies needed to support students with these challenges as a priority if they expected students to engage in learning. The final response category was a perception of student health as important in its own right (as opposed to simply a determinant of attainment) and that academies had a duty to ensure students had the knowledge, skills and opportunity to lead healthy lives. Respondents with this view were again drawn from all levels of the trust hierarchy. Table 3 shows the response categories and some illustrative quotes.

**Table 3:** Academy trust respondents: the role of schools in student health promotion

Response category	Quotations
No responsibility	*Fundamentally, we are charged to educate children. Whilst we clearly have a strong interest in promoting health that is not actually our core business. It’s not that we don’t want to do it. It’s just the available time and capacity and resource. (AS1)*
Functional approach	*Healthy children will have fewer absences and absences are linked to GSCE grades.* *They have to be here to learn. (AS2)*
Removal of barriers to learning	*Unless we can remove those barriers to learning, they are not going to access the curriculum. They’re not in an emotionally sound place, they’re not feeling secure enough. (AS7)* *If you have healthy, happy children they hopefully will go on to have the better potential to attain. (AS8)*
Duty to promote good health	*There is [no point] being successful, academically, if you have a short life span. It’s a bit of a pointless exercise, so our conclusion we have come to is that, actually, the most important thing for these children is their wellbeing. (AS13)* *There is a big commitment in the trust to ensure young people are fit and healthy, because we recognise that that is a driver to them being happy and successful (AS10)*

**Fig. 1:**
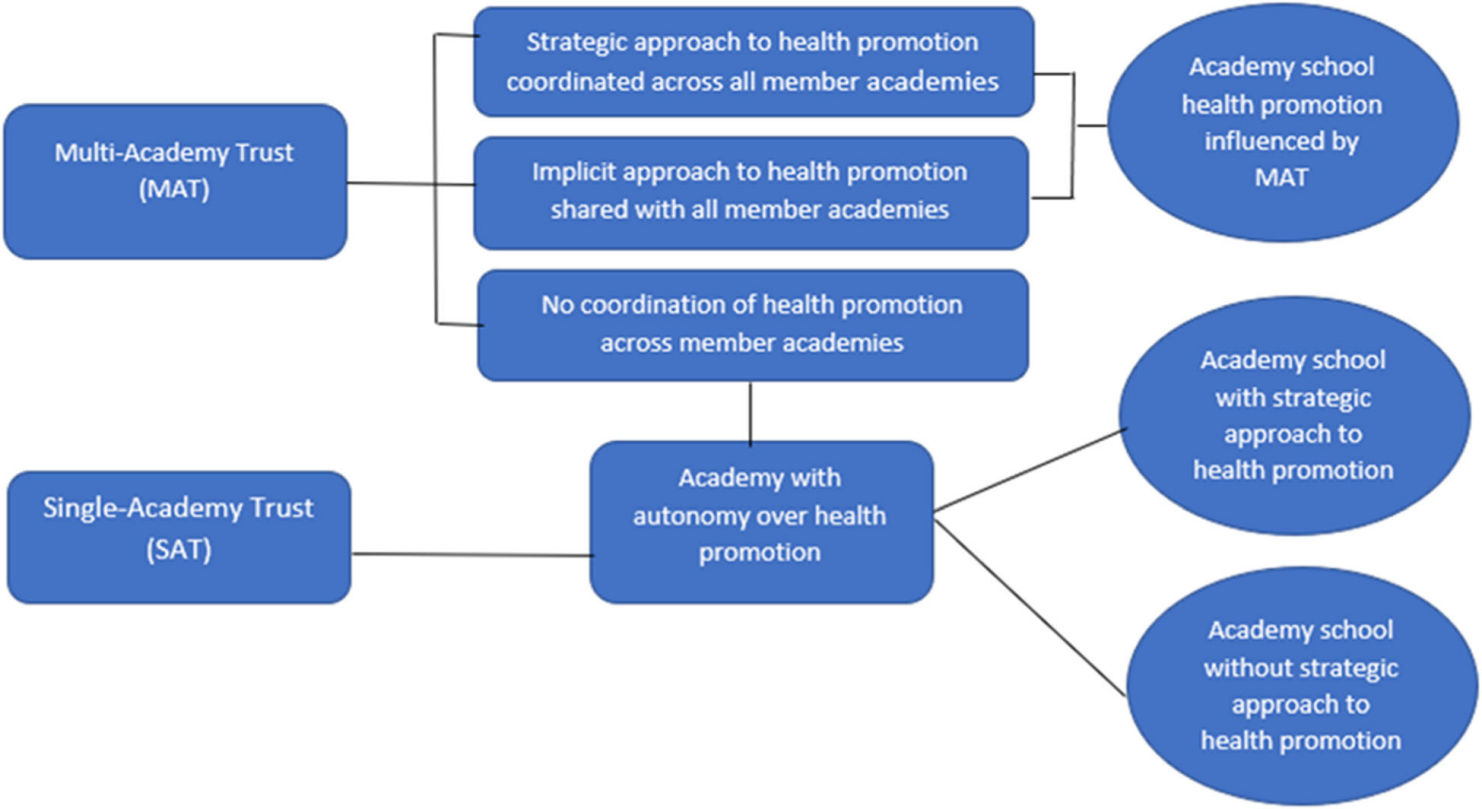
Variability in approach to health promotion across MATs and SATs

The range of views on the role of academies in health promotion was reflected in their organisational responses to the health challenges facing students. Figure 1 shows how academy schools varied in their approach to health promotion depending upon whether they were part of a MAT, or a single academy trust.

Most MATs in the sample did not have a centralised approach to health promotion and individual academies within the trust retained autonomy in this area. Some had developed trust-wide strategies. There was no association apparent in the data between the size of trust and the approach taken.

Given the agreement among respondents of the importance of student mental health, this topic is used here to illustrate the varying approaches.

#### MAT-wide strategic approach coordinated across all member academies

There were examples in the data of MATs which had developed a strategic approach that was being implemented in all academies within the trust, with funding allocated from both a central MAT budget, and an expectation that some resource from individual school budgets would also be used. Some of these trusts had invested in professional staff including educational and clinical psychologists to provide support to implement whole-school approaches as well as targeted support to individual students. In some cases, this was funded from a pooled central budget; in others, academies in the trusts paid for packages of support from their individual budgets. One MAT in the sample had invested in mental health training for the majority of teaching staff in the trust and had directed each academy to develop a bespoke mental health curriculum. A key commonality in this approach was a trust policy for student mental health developed at the executive level and implemented by schools. This approach was seen in both small to medium MATs, the federation, and one of the larger MATs.

#### MAT-wide implicit approach shared with member academies

In contrast, there were also MATs which did not attempt to (formally) coordinate mental health support for students across the trust, but CEOs or other members of the executive team would ‘*set the tone and direction’* (AS11). A number of CEOs of trusts within this group stated that the approach was ‘*implied*’ or ‘*agreed*’ and academy leaders would be expected to implement strategies that complied with the CEO’s view, but no written strategy existed, or central funding allocated.

[Initiatives around health are] *decided by the school. I give the top level steer. “We need more focus on this. You need to do it through assemblies, through visiting speakers, through health education in dedicated PSHE time. And you need every adult in the school to understand it so that it can be woven into other transactions as well.” They’re the key messages. And I ask them to tell me then what they’re doing.(AS9).*

The analysis also identified that MATs with a coordinated approach (explicit or implicit) often approached aspects of health differently; there existed trust-wide strategies that focussed only on student mental health, or only on physical activity, and one that attempted to identify good practice in all aspects of student health and wellbeing and standardise this across all members schools.

#### MATs with no coordinated approach

Responses indicated that most MAT’s executive teams did not attempt to determine any strategic MAT-wide approach, explicit or implied. In the main, most academy schools retained autonomy over health promotion regardless of MAT or SAT membership.

Senior executives from these trusts with no coordinated approach stated clearly that decisions around mental health support were taken at the academy level. In some cases, executive level staff interviewed were unable to describe the types or initiatives implemented by member academies as this information was not collated or shared across the trust. Again, this approach was seen in trusts of all sizes, and matched with the experience of non-education respondents.

*We, kind of, naively thought, “Multi-academy trust, academy chains, it’s going to work exactly the same way [as local authority education departments]. We’ll go to them, we’ll explain, they’ll see the value of this, and, you know, instead of working with local authorities, we’ll work with MATs.” It just hasn’t been like that at all. We’ve had lots of meetings with academy chains that, kind of, apparently get it, are really interested. All bar one or two have then come to nothing. (NS23).*

This was supported by a local authority public health officer who perceived the engagement of individual schools was mediated by their history of engagement prior to converting to academy status, rather than by the MAT executive:

*I feel that the relationship we have with our current schools, I think, is very largely based on the relationship we had with them before they became academies…[]… What’s happening [here], which is a lot of schools engaging with us doing all sorts of different things, isn’t about it coming from the [trust], it’s coming from us and the relationship we shared. (NS28).*

Some trust leaders attributed the lack of centralised strategy to the age of the trust, with more recently-formed MATs still developing the role of the executive team.

#### SATs and academies within MATs with a strategic approach

Respondents from SATs, and from MAT academies where the trust executive did not attempt to coordinate or influence health promotion in member schools, were clear that academy head teachers were responsible for how health promotion was prioritised (or not), what strategy was implemented, and how much funding allocated. This group comprised schools that had developed explicit mental health policies or strategies and invested some resource in implementing initiatives to support students. Often these schools had a named lead for student mental health. One SAT respondent described allocating considerable resource in the development of a specialised ‘mental health base’ providing on-site, long-term targeted support for students, with three full-time staff in place. Other schools had similarly invested in specialised staff, most often counsellors, school nurses, and family support staff. Respondents also talked about investing time and resource in appointing and training dedicated (i.e. non-teaching) pastoral support staff. Other initiatives implemented by academy schools included the use of standardised measures to measure wellbeing or identify vulnerability, and using external organisations to deliver elements of the PHSE curriculum and/or additional extra-curriculum activity intended to promote wellbeing. Respondents also gave examples of taking part in trials of interventions intended to support student mental health, coordinated by external agencies perceived as expert in this area.

#### SATs and academies within MATs without a strategic approach

There were also a small number of responses that indicated some academies had no identifiable mental health strategy or allocated funding. Respondents described some activities they considered *may* impact on student wellbeing including the availability of pastoral support, extra-curriculum provision that included yoga and physical activity opportunities which respondents believed would impact on wellbeing. However, we could perceive no indication from participant responses or the review of publicly available information about the school that this was part of a coordinated or targeted approach to promoting good mental health, or delivering targeted support initiatives for students with poor mental health.

### Research question 2: how are decisions around health promotion made in academy trusts?

Respondents from academy trusts were asked how decisions about health promotion were taken in the trust, in particular around whether or not to influence individual academy schools or allow them autonomy. None of the respondents at trust executive level cited accountability to state agencies such as Ofsted (the government agency that inspects all maintained and academy schools in England) or the Department for Education. Within MATs respondents who indicated that the executive team did influence health promotion within academies, either through an explicit strategic approach or through the implicit influence of the CEO and/or executive team, were largely in agreement that this was determined by the CEO. CEOs who had prioritised health promotion attributed this to their own *‘special interest’* rather than any external impetus, and the existence of a trust-wide approach was largely dependent on whether the trust CEO preferred this approach:

[The health strategy is] *centrally led by the trust, without a shadow of a doubt. It comes from me, my executive team. So, things like the mental health first aid training, there is absolutely no way we would have trained [so many] teachers last year if it had been left to the individual schools to make decisions. (AS25).*

*This is where we differ from other trusts, I like my schools to be autonomous. I have got some superb head teachers and I don’t want to constrain them by saying, “It’s Monday and you will do this.” I want them to be free to be able to do the things they want to do. (AS22).*

One respondent was cautious about the potential dangers of trust CEOs having autonomy over MAT’s approach to health:

*Mental health is something that I’m very alert to, and I have the privilege of being the CEO of a multi-academy trust, but there are other CEOs who couldn’t care less. And they’d say, “Well why should they?” You know, “We’ve got to get results.” So that contributes to a very complex landscape…[]…That’s part of the problem with MATs, you get CEOs who really kind of fancy themselves, they say they’ve got all the answers, but they don’t, they don’t test it.* (AS26).

### Research question 3: what factors inhibit and encourage health promotion in academy schools?

While most trust leaders did not feel accountable to the UK Department of Education or Ofsted around health promotion, other factors emerged that influenced activity. Factors that inhibited health promotion in academy schools included financial constraints, the prioritisation of educational outcomes, a perceived lack of accountability for health promotion, and limited understanding about which initiatives might be most effective.

Many respondents noted that school budgets were tight, and most schools did not have a ring-fenced budget for health-related activity. In addition, head teachers facing difficult choices would prioritise maintaining teaching staff levels, which in some cases meant cutting health-related provision. Examples of services cut because of financial constraints included counselling and nursing, as well some extra-curriculum enrichment activity. Respondents also noted that the unpredictability of future budgets meant they were reluctant to invest in any new initiatives, health or otherwise, because they could not guarantee their sustainability.

School staff noted the pressure to prioritise student educational outcomes. At primary level, standardised attainment tests (SATs) are undertaken in Years 2 and 6 in English reading, English writing and mathematics, with schools set targets for the percentage of pupils achieving the ‘expected standard’ in Year 6. At secondary level, important outcomes include GSCE and A-level grades, and Progress 8, a measure of students’ progress across eight subjects. These measures were perceived as the key indicators of school performance and head teachers were under extreme pressure to maintain or improve them. This led to a focus on these outcomes often at the expense of other activities, particularly where schools were under pressure because of previous poor performance.

*Any changes that would make a difference to health would have to be weighed against the effect it would have on the school’s standing, and how we’re perceived out there, the exam results and so on. If that could be made neutral, then we would be willing to do that, …[]… We had a couple of years before this last tranche of targets and bits and pieces where we were hovering at the base, you know, 40%, five A-Cs which, if you went below that, you were in serious trouble. …[]…. But we’re in a stronger position now where we can say, “You know what? Let’s take the hit on a couple of… Because it would be so much better for the kids. The kids will be happier, or the staff will be happier.”*(AS4).

The fear of a poor Ofsted assessment result was common across all education respondents, and the impact this had on the low prioritisation of health promotion was exacerbated by the perception amongst most respondents that health promoting activity did not contribute to a good assessment outcome. While the Ofsted inspection framework includes criteria referring to students’ knowledge and skills about staying healthy both physically and mentally, few respondents perceived that Ofsted inspectors focussed on these criteria but rather on pupil attainment, behaviour and attendance rates.

Head teachers and wellbeing leads within academies also described difficulties in finding information about health promotion, and in understanding what initiatives might prove effective. For some this was because of a belief that reliable resources would have costs associated with them, which they could not afford. Others reported that there was often too *much* information available, often free online, but which they lacked the skills and time to navigate effectively. This resulted in staff feeling they lacked the expertise to address health concerns in their schools, and an unwillingness to invest resource into initiatives that may not work. Some respondents were also unclear about the schools’ relationship with statutory services:

*A couple of years ago, at board level we looked at whether we should have a school nurse pilot across all of our schools. That really came from our chair of the board essentially saying, “Obesity is a massive problem. What’s happening in our schools to address this?” I spoke to quite a few local authorities, and obviously the [health care professionals]… “Obviously, our kids get screened in Reception and Year 6. All of those things currently happen. But what more could we add?” At the time we didn’t move forward with that, because there were complications around funding or complications around who we would employ to do it. “Where would this sit against what the local authority…?” It was just quite murky. There is a lot of appetite at our board level to do something, but it’s just finding out the what. They would fund it, but it’s finding out where we would get the biggest bang for the investment.* (AS21).

There were also factors emerging from the data that encouraged health promotion in academies. Respondents working in schools sited in areas of high socio-economic deprivation often talked about being compelled to address student health because of the difficulties children and young people were facing. These included extreme poverty, and several respondents in the sample worked in academies that regularly signposted children and parents to food banks, with some also reporting ‘staff whip-rounds’ to buy food and clothes for pupils’ families in emergencies. Other community difficulties included high rates of crime, substance misuse, domestic and gang-related violence, all of which impacted on student wellbeing. Respondents were conscious that wider austerity measures had resulted in cuts to health and social care services which limited the support available to students outside of school. This prompted some respondents to prioritise wellbeing as *more* important than attainment, and also because students could not access the curriculum until their health needs were met:

*So, we get the most challenging and chaotic students in this academy. So, we know that we’re not going to get the best results, which is a shame, because we put massive amounts of work and effort in, and the teaching staff are amazing, but I think because there’s so much external issues, it has a massive impact on the students. So, we have to put a lot of nurturing, parenting in before we get anywhere close to them in achieving with them…[]…For me, our students’ emotional wellbeing, health, and their safety is paramount, over a GCSE.* (AS6).

This view was supported by non-education respondents:

*With so many services going, particularly around health and wellbeing, schools have to skill themselves up. They’re desperate [for training] because they know they’ve got to work in this area.(NS28).*

Mirroring the influence of trust CEOs over the approach to health in MATs, head teachers were frequently cited by respondents as key to determining how health promotion was addressed in autonomous academy schools. Several gave examples of how the resource dedicated to health promotion (including core staff time, staff training, equipment, and funding for external agencies to deliver health-related initiatives in schools) was entirely within the remit of the head teacher to determine, and that these resources could expand or contract quite dramatically with a change of head teacher. Non-education respondents confirmed that in their experience, the prioritisation (or not) of health was determined by the head teacher.

*There was one school I worked with that had three or maybe even four headteachers involved over the course of a year because of special measures, temporary people and so on. The staff morale around teaching PSHE* [personal, social, health and economic education] *and RSE* [relationship and sex education] *was very low. It wasn’t prioritised…[]… So, although the head teacher said they support it, they weren’t actually giving it enough attention or resource. …[]…Until they’ve got some leadership that’s helping them across the board, they’re not going to make a massive transformation.* (NS29).

Head teachers themselves agreed with this, albeit they also noted that budgets would be overseen by the MAT executive (where applicable) and school governance or advisory boards would also need to agree.

Head teachers were more likely to prioritise health promotion if they believed that student health and education outcomes were linked. Most heads agreed that they were, but there appeared some association between those who perceived poor health as a barrier to learning and allocating dedicated resource to student health.

*The barriers pupils are facing are significant. Unless we can remove those barriers to learning, they’re just not going to access the curriculum in the same way. They’re not in an emotionally sound place, they’re not feeling secure enough, their behaviour is potentially challenging, they may be suffering anxiety or concern outside of school. For us, removing the barrier to learning is absolutely essential. (AS7).*

Academy schools often worked in partnership with external organisations they perceived as experts in health. Partners included statutory health services (e.g. Child and Adolescent Mental Health Services (CAMHS)), third sector organisations and for-profit health organisations. School staff frequently talked about using external organisations to deliver aspects of the PHSE curriculum. It was not always clear how schools assessed the quality of this provision, beyond relying on word-of-mouth testimony from colleagues in neighbouring schools. Free or low-cost initiatives were the preferred choice. Some schools had been involved in pilot initiatives developed and evaluated by external partners. Most commonly these focused on mental health and wellbeing and partners included the Anna Freud National Centre for Children and Families, and CAMHS. Few schools reported contact with or support from local authority public health teams but where school did have this support, it was greatly valued.

*I think [this city] is one of the only areas that have the Healthy Schools team now. They are a very strong team, although very under-funded. They support us with the curriculum. They are responsible for five key areas, so things around diet and lifestyle, emotional wellbeing, mental health, sex and relationship education, drugs and alcohol. They lead, really, in terms of looking at current research, developing materials.*(AS14).

Respondents from local authority public health acknowledged that the loss of national co-ordination of the Healthy Schools programme (supporting schools to achieve ‘Healthy School’ status across four areas; Personal, social, health and economic education (PSHE), healthy eating, physical activity and emotional health), and cuts to local authority public health budgets, had affected their offer to all schools, including academies.

*We fought, myself and one of our public health consultants, yes, we did unsuccessfully fight to retain Healthy Schools [in this area]. …[]… The offer we have for schools around the public health agenda is poor…[]…I wouldn’t say there is a consistent level of input to a public health framework into our schools, nor is there a consistent knowledge of what our schools are doing because they are not accountable to us in any way, shape or form. (NS20).*

### Research question 4. How might public health academics and practitioners best engage with academy schools to facilitate health promotion activity and research?

Education respondents all expressed interest in being involved in research on health promotion amongst both staff and students. Few had had previous experience of working with academia in this way although a small number had been involved in the piloting and evaluation of health-promoting initiatives. Table 4 summaries participant’s recommendations for undertaking new health-related research in academies.

**Table 4:** Recommendations for undertaking research in academy schools

Early engagement	• School input into identifying evidence gaps and research aims • School input into methodological design • Long lead time for consultation with staff, pupils and parents • Fit with school planning cycles
Minimal impact	• Avoid or reduce disruption to student curriculum • Avoid crucial points for students e.g. exam time; pre- or post-transition • Minimise impact on staff workload • Payment for staff release if required • Avoidance of disruptive change of any type
Tangible benefits for schools	• Contribution to curriculum, and curriculum development • Contribution to school health strategy • Continuing Professional Development (CPD) opportunities for staff • Development of resources with practical implications of use to schools e.g. staff training resources; teaching and curriculum resources • Development of data of interest to schools (benchmarking) • Avoidance of data presentation that may stigmatise communities (e.g. poor health data) • Offering widening participation opportunities to school pupils • Building an ongoing relationship between the school and University
Outcomes of interest	• Research aims aligned with school curriculum • Research aims aligned with school health priorities or challenges • Clear links to attainment outcomes

### Stakeholder event

Feedback on a presentation of the study findings from a group of 40 stakeholders from academia, local authority public health teams, and third sector organisations with experience in working with both maintained and academy schools indicated that they resonated strongly. Delegates agreed with the main finding that health has variable status in academy trusts:

*“In [City Y] we have a high number of MATs. Although the Trusts promote a trust-wide intention, the final decision always sits with the school. Deprivation and local inequalities drive their decisions.” (*event delegate*).*

Delegates also confirmed that promoting the link between health and attainment was key to engaging academies in both health promotion and health research. More detailed feedback from the event is provided in an additional document (Additional file 3).

## Discussion

This study revealed wide variation amongst senior academy and trust leaders in how they perceive the role of academies in promoting wellbeing amongst students. Education respondents all agreed that promoting good staff health was important for the reduction of staff absence and long-term staff retention. Student mental health was identified as the key challenge. However, there was little agreement on the role of academy schools and trusts in responding to this challenge. It is encouraging that few respondents suggested that academy schools had no responsibility for health promotion amongst both students and staff. More prevalent in this study was the perception of health promotion as a means of reducing pupil and staff absence, and improving staff retention. Some perceived health promotion activity as essential to the removal of barriers to learning amongst pupils and subsequently raising attainment and school performance outcomes. There were also respondents in the current study who believed that academy schools should prioritise health for its own sake rather than simply as a means of raising attainment.

There was a lack of agreement between MAT leaders over whether member academies should be directed, or maintain autonomy, over student health promotion. Most MATs do not have a centralised approach and individual academies retain responsibility for this area. This was seen in trusts of all sizes and resonated with the experience of non-education respondents trying to work with MATs to promote health. Where we found MATs that did attempt to coordinate (some) health promotion, this was entirely dependent on the CEO’s attitude and interest in health promotion rather than any perceived external accountability. Some MAT leaders attributed this limited attention paid to health as a symptom of many trusts being in their early stages of development and suggested that health may be an area that would receive greater focus on the future. Indeed Ehren and Godfrey’s case study of one MAT revealed how the trust initially focussed on operations and finance, before turning their attention to curriculum, assessment and school improvement [11]. It may be that as MATs develop, health promotion may be more likely to be included as part of a centralised school improvement strategy. Meanwhile most academies (within MATs or not) retain autonomy over health promotion and head teachers determine the resource dedicated to it.

The barriers and facilitators to health promotion in academies identified by this study are important for research and practice within England and globally. Financial constraints, a focus on educational outcomes and school performance, and limited understanding of which health initiatives might be effective and hence worth investment were all cited as key barriers to prioritising health promotion. Conversely, academies which do prioritise health often do so because the health inequalities amongst students (both within and across academies) are stark; because school leaders perceive the impact of poverty, and high levels of crime, substance misuse, domestic and gang violence in the communities in which they are sited means that student health is severely challenged and must be prioritised; that these challenges must be addressed before students can access the curriculum, that austerity measures mean that students may not be able to access support outside the school; and/or because school staff are supported by ‘expert others’ and therefore have more confidence in the efficacy of health-related initiatives.

This last factor suggests that academies would welcome support from public health partners. Where academies had the opportunity to work in partnership with external agencies to deliver health-related initiatives, they often did so. Preferred partnerships included those perceived as expert in health, including CAMHS and local authority Healthy Schools teams, who could support schools in identifying and implementing evidence-based initiatives.

Ofsted inspections dominated participants’ accounts of the external accountability of academies and MATs but few participants perceived that Ofsted placed much emphasis on health promotion. This may change; a new Ofsted Inspection Framework is intended to refocus school inspections on curriculum content and overall quality of education and includes modest new health-related criteria [24, 25]. New developments in health education in England are also forthcoming. Driven by concerns about the level of provision and quality of sex and relationships education (SRE) in schools, the UK government enacted legislation in 2017 requiring all school to provide SRE [26]. The Department for Education is introducing mandatory Health Education in all state funded schools, and draft guidance for this provision indicates that the curriculum will include mental wellbeing, internet safety, physical health and fitness, healthy eating, drugs, alcohol and tobacco use, prevention of poor health, first aid, and body development in addition to SRE. These will be taught in both primary and secondary schools (sex education will only be addressed in secondary schools) [27].

All education respondents in this study were interested in undertaking new research on health promotion in schools, thereby highlighting an interest in school-based research. Their recommendations for facilitating this included the early engagement in the research cycle of academy and trust staff affording them the opportunity to influence research aims and ensure they were aligned with school curricula and health priorities as well as educational outcomes. Education respondents also want research outputs to include those with tangible benefits for schools such as improvements in educational attainment, reductions in disruptions and positive impacts on test scores.

This study is limited by the lack of comparison with maintained schools and it is possible that some of the barriers to health promotion identified in academy schools is common to all school types. However, delegates at the stakeholder event agreed with the account that academy schools and trusts do not, as yet, have a consistent approach to health promotion and are often more difficult to engage with than local authority-maintained schools. As the academies programme continues to expand in England, understanding how health promotion is undertaken in these schools will remain an important issue for public health researchers.

## Conclusion

In the current absence of national policy or guidance around health promotion in schools, together with reduced influence of local authority healthy school teams, health has variable status in academies in England. This matters, as over half of all students attend an academy school. There is a need to better engage **all** academy trusts in health promotion to ensure equitable provision for students. Assessing the impact of forthcoming mandatory Health Education, and a new inspection framework, on pupil health and health attitudes will be an important public health and research issue. Public health academics and practitioners should better communicate the links between health and educational attainment outcomes to academy leaders and support academies to implement a strategic approach to health promotion.

## Additional files

Additional file 1: Glossary of school terms in England. (DOCX 14 kb)

Additional file 2: Topic Guide. Guide for qualitative interviews. (DOCX 20 kb)

Additional file 3: Stakeholder Engagement Event. Qualitative feedback from initial dissemination of early findings. (DOCX 21 kb)

## Data Availability

The qualitative datasets generated and/or analysed during the current study are not publicly available due to the data containing information that could compromise research participant privacy/consent but are available from the corresponding author on reasonable request, and subject to approval from the School for Policy Studies ethics and research committee at the University of Bristol.

## Abbreviations

AS: Academy school
CAMHS: Child and Adolescent Mental Health Services
CEO: Chief executive officer
CPD: Continuing professional development
MAT: Multi-academy trust
NHS: National Health Service
NS: Non-school (participant)
Ofsted: Office for Standards in Education, Children’s Services and Skills
PRU: Pupil referral unit
PSHE: Personal, social, health and economic education
SAT: Single academy trust
SATs: Standardised attainment tests
SEND: Special educational needs or disabilities
SRE: Sex and relationships education
