# Supplementary material for: A qualitative study of health promotion in academy schools in England
Source: BMC Public Health. 2019 Aug 28;19:1186. doi: 10.1186/s12889-019-7510-x (PMC6714088; doi:10.1186/s12889-019-7510-x)
Supplement: Supplementary file 1 — Glossary of school terms in England. (DOCX 14 kb) [file 12889_2019_7510_MOESM1_ESM.docx]

**Additional File 1. Glossary of school terms in England**

**Maintained-school: Funded and controlled by the local education authority.**

**Academy**: Publicly funded independent school, run by an Academy Trust. Academies are funded directly by national government, not the local authority. Some academies have a sponsor, such as a business, university, or other school, who has majority control of the academy trust and whose role is to improve under-performing schools. Other (higher-performing) schools are allowed to convert to academy status without a sponsor to gain increased autonomy.

**Single Academy Trust (SAT) -** A single academy trust runs one academy.

**Multi Academy Trust (MAT) –** Multi-academy trusts (MATs) run more than one academy. Currently the largest of these in England are responsible for over 60 academies, and cross multiple local authority boundaries.

**Free school**: These are also academies, but is the term used for new schools set up independently of local authority control. They can be set up by groups such as charities, universities, faith groups or Academy Trusts.

**Special school**: Cater for pupils aged 11 and older and specialise in provision for children with special educational needs.

**Faith academies**: like other academies but may have their own admissions process, and can choose what they teach in religious studies.

**Pupil Referral Unit (PRU):** Provides education for children who are excluded, sick or otherwise unable to attend mainstream school.
