# Supplementary material for: A qualitative study of health promotion in academy schools in England
Source: BMC Public Health. 2019 Aug 28;19:1186. doi: 10.1186/s12889-019-7510-x (PMC6714088; doi:10.1186/s12889-019-7510-x)
Supplement: Supplementary file 2 — Topic Guide. Guide for qualitative interviews. (DOCX 20 kb) [file 12889_2019_7510_MOESM2_ESM.docx]

**Additional File 2: Topic Guide**

**Engaging with academy and free schools to enable evidence-based health promotion in schools**

Note: This topic guide is indicative

**Introduction:**

- Thank you for participating
- Introduce self and NIHR SPHR
- Introduce the study
- Talk through key points:
- length of interview
- interview like a discussion, but will cover key topics
- no right or wrong answers
- participation is voluntary, rights to withdraw
- recording interview (concentrate on what you are saying, accuracy)
- Confidentiality and anonymity, how findings will be reported
- Questions?
- Happy to proceed? Sign consent form

**START RECORDING**

**Attitudes and beliefs towards health promotion in schools**

- Do you think that there is a link between health and learning (education attainment)?
  - What is the nature of the association?
- Which health domain is of most interest to you and why?
- How well informed do you feel about the evidence of what works in schools to improve health and wellbeing?
  - Students
  - staff
- What sources of information / evidence do you have about what works in schools to improve health and wellbeing?
  - Where does this information come from?
  - How good is it?
  - What information/evidence would you find useful?

**Health promotion initiatives/programmes in schools**

- Where does health fit in the strategy of the Trust / individual schools?
  - Does any one individual or committee lead on health?
    - If yes, who, and role
- What is the extent to which student and staff health and wellbeing features in strategic planning?
  - How does the academy chain / alliance affect decisions at the individual school level
- Who makes these decisions
  - Trust board, local governing board, head teachers, other?
  - How are decisions taken at the trust level communicated to schools
  - How are they implemented?
- Thinking about the differences between maintained and Academy schools, what do you think has been the main difference to your role since leaving the local authority?
  - And what difference has leaving the LA made to health promotion?
- To what extent is health taken into account in wider decision-making, for example changes to school buildings/new buildings;
- Thinking about improvements in student and staff wellbeing, how far would you be prepared to go in making changes to
  - the curriculum,
  - the school environment,
  - the school ethos
  - the relationship between staff and pupils, and
  - the way schools interact with parents and the local community?
- Does the school participate in any healthy schools programmes or use the healthy schools audit?
- What budgets do you have (how much is currently spent on health promotion)?
  - If MAT, how are these budgets allocated to individual schools?
  - What is the source of these budgets?
  - Do they have any rules around expenditure and how do you navigate those rules?
    - Pupil premium
    - Apprenticeship levy
    - Sports premium
- Has the Trust/school recently undertaken any activity or intervention in
  - Physical activity
  - Nutrition
  - Risk behaviours including drugs and alcohol
  - Sexual health
  - emotional wellbeing and mental health
  - violence/bullying
- Are there any pupil-led initiatives?
- Who currently delivers the programs (training school staff versus external providers)?
  - What are the pros and cons of each approach?
  - What would you prefer?
- Does health and wellbeing feature in teacher training/inset days? How is the agenda for these decided?

**Health improvement research in schools**

- Do you collect data on health?
  - For primary schools, do they use data to track 30:30?
- If yes, what is collected?
- What is the quality of data?
- Would you share anonymised data with researchers?
- When in the school day/week best for data collection?
- Which evaluation frameworks would be suitable/practical?

**Engaging with public health practitioners or academics**

- Do you already work with public health practitioners or academics to improve health and wellbeing?
- If so, describe
- What works well, what doesn’t
  - How could things work better?
  - Would the school / collaboration / Trust be interested in being part of a school research network?
  - What would influence decision making?
- If not, would you be interested in joining forces with public health academics and practitioners to improve the health and wellbeing of your staff and students?
- What mechanisms could be put in place to facilitate this?
- How do we frame the working relationship (how can we help you?)

**Closing**

- Thank you
- Any questions?
- Copy of interview transcript?
- Summary of results from the study?
