# Supplementary material for: A qualitative study of health promotion in academy schools in England
Source: BMC Public Health. 2019 Aug 28;19:1186. doi: 10.1186/s12889-019-7510-x (PMC6714088; doi:10.1186/s12889-019-7510-x)
Supplement: Supplementary file 3 — Stakeholder Engagement Event. Qualitative feedback from initial dissemination of early findings. (DOCX 21 kb) [file 12889_2019_7510_MOESM3_ESM.docx]

**Additional file 3 Stakeholder Engagement Event**

Early findings were presented to 40 stakeholders from academia, local authority public health teams, and third sector organisations with experience in working with both maintained and academy schools. Consent was gained to collate written, anonymised feedback from the event on delegates’ agreement (or otherwise) with the findings.

**1. Whether findings resonate:**

- *To some degree these finding echo my experience of interacting with academy schools. However, the academies I have engaged with had no/very limited concerns for the health of their students, so I think their responses are likely to have been even more demoralising than your findings! (I suspect they would have been vanishingly unlikely to participate in such research in the first place – would be keen to read your discussion of selection bias in this study!)*
- *Definitely resonates with my experience*
- *Yes, the study findings resonate with my experiences – there are difficulties in delivering Health Promotion in the academies due to their packed curriculum*
- *In [City X] we have a high number of MATs – although the Trusts promote a trust wide intention as you said, the final decision always sits with the school. Deprivation and local inequalities drive their decisions. Mental Health is a priority for schools. Some primaries did an RCT– they wanted impact on attainment and other research needs as you stated*
- *More or less. I work on [intervention] so in a different context. There, the health promotion is made by health centre’s workers.*
- *We are charity providing health education resources to schools. I would agree with the study’s findings that it is very difficult to find the appropriate person to speak to within academy chains (and sometimes individual academies!)*
- *Our health promotion in schools award asks who at governance level is responsible for health and wellbeing? More schools name this person easily. Recently a small MAT school did not know the person at Trustee level which shocked me.*
- *Headteachers opinions and beliefs often paramount to direction of schools’ “health”. Both hinder and drive forward.*
- *The findings certainly resonate with the areas I have worked in however in particularly:*

*-Schools focus on attainment*

*-Confusion around contacts*

*-Finances*

*-Mainly the links between attainment and health*

*-CPD for staff*

**2. Barriers and facilitators to engagement with schools**

**Facilitators to engagement with schools**

- *I have noticed some schools will openly welcome health support and make their own decisions, whilst others state they can’t make decisions without speaking to sponsors – not a priority.*
- *Schools and local authority education groups really welcome having a health consultation to inform discussions, signposting and health information/policy to promote their understanding and can be applied in their setting rather than focus being on a project or teaching delivery level.*
- *Link to attainment made a priority -attainment is often the key to conversation and can then look/discuss health/attainment link.*
- *The easier you make it for schools to implement and less pressure you place on school staff the more likely they are to engage with your service*
- *Academies will engage if research could indicate their academy ethos and approach results in (inc Health work) significant differences and impact on pupil attendance and achievement.*
- *Local partnership work with CAMHS has encouraged schools to engage with mental health whole school approach programme. If a key member of staff leaves can be hard to keep the work going.*

**Barrier/difficulties**

- *Headteachers with a personal interest in physical or mental health are more engage with our services. They understand the importance from their own experiences and are keen to promote health and wellbeing across their school. Feels like schools are being left to do this on their own. Need to ensure joined up working between health providers and education providers.*
- *One of the barriers in my opinion is that schools are often approached for research and get little in return – especially one off studies. They need personalised reports, Ofsted and behavioural focus etc and we work successfully on longitudinal studies with lots of reports for schools instantly available online.*
- *Staff and student health and wellbeing is being considered more but budget dictates otherwise.*
- *Lack of the recognisable healthy schools awards/packages (now all funding has gone and school improvement offers have been lost) has had a massive impact on schools interest in health.*
- *Health delivery and capacity/resources limited in secondary so often subpar.*
- *Funding challenges are having a direct impact on schools being able to send staff on training. Even if training is free, they can’t afford the cover.*
- *In [county X], we are fortunate enough to offer a “Healthy Schools” Programme for free to all schools, however only 20% of schools engage with us, so money is not the main barrier, we found time and priority for attainment and attendance are our main barriers.*
- *In a small authority relationships with MATS etc still strong. Issue many changing heads. Schools always keen to take health interventions where money is provided, however not always considered in terms of the whole school approach. Surprisingly back in the day of the National Healthy Schools Programme 97% of our schools achieved status!*
- *Increasing challenges re financial restrictions which appear to be restricting schools’ capacity to engage in training/programmes that support subject teaching and attainment not JUST those that have more health focus. There is interest and willingness for health but limited as result of resource primarily .*
- *School pressures and not recognising health promotion as valuable to attainment and school time all common themes within [City] and trying to engage schools. We are much better at engaging primaries rather than secondaries.*
- *Very hard to find anyone at schools who is the logical/interested contact point for health related. It feels like no schools’ staff have any spare time/mental bandwidth.*
- *Definitely a struggle getting the right person in the school at the right time. Can show an interest but then have to get decisions approved, organise workload – other priorities take over.*
- *If an obesity intervention in schools if the headteacher or someone in the leadership team have had or currently having a “weight issue” this is a huge barrier with engaging in the school.*

1. **Evidence**

- *Concerned that the evidence base from Public Health England is not setting to our interventions practice.*
- *As a school nurse I am constantly battling with schools to acknowledge the links between health and attainment. The main issue identified being the impact Mental Health is having on young people accessing school and social isolation.*
- *Interested to see that the feedback was a want for evidence. In my experience locally schools sometimes/often miss this! As a PH team we encourage them to look for evidence and impact but they often engage with novel/well marketed/low cost/low workload intervention. These can also often be unsustainable.*
- *Link between physical health and mental health not always understood. Siloed to treat separately.*
- *Schools saying that “if the evidence was there that it ticks boxes for Ofsted, raises attainment and reduces absenteeism, that would make a difference”. In fact the evidence base is there that PSHE does all of these, and has been for many years – that’s the message we need to get into schools.*
- *Being from a school the hesitation around evidence based is definitely more around the mental health agenda.*
- *We find in our area it has become even harder to engage with secondary schools (on any topic) since they have become academies.*
